# Psychological Aspects and Implications of Food Addiction and Glucose Control in Type 2 Diabetes: A Pilot Mixed-Methods Study

**DOI:** 10.3390/healthcare14040420

**Published:** 2026-02-07

**Authors:** David J. Johnson, Laura A. Buchanan, Erin M. Saner, Matthew W. Calkins, Julienne K. Kirk

**Affiliations:** 1 Department of Family and Community Medicine, Wake Forest University School of Medicine, Winston-Salem, NC 271571, USA; 2 Toward Health, Blauvelt, NY 10913, USA; 3 One Health, Rural Hall, NC 27045, USA

**Keywords:** continuous glucose monitoring, group medical visits, food addiction, patient activation

## Abstract

**Background/Objectives**: Type 2 diabetes (T2D) affects more than 38 million Americans and remains a leading public health challenge. Behavioral self-management is central to glycemic control but is often undermined by dysregulated and addictive-like eating behaviors. Continuous glucose monitoring (CGM) offers immediate feedback that may strengthen self-regulation, yet the psychological processes linking CGM use, food addiction (FA), and behavior change are poorly understood. This secondary mixed-methods study examined how CGM-supported group medical visits (GMVs) influence glycemic outcomes and FA symptoms in adults with diabetes. **Methods**: Adults with T2D participated in a 14-week GMV program integrating CGM review with education on nutrition, physical activity, sleep, stress, and intermittent fasting. Thirteen participants had paired CGM summaries and psychosocial data. Quantitative outcomes included mean glucose, glycemic variability, time-in-range (TIR), and symptoms of food addiction using the modified Yale Food Addiction Scale 2.0 (mYFAS 2.0). Qualitative data came from open-ended surveys analyzed using reflexive thematic analysis. Integration followed a convergent design, merging individual change trajectories with thematic interpretations and case vignettes. **Results**: Mean glucose decreased by 21 mg/dL and TIR improved by 9 percentage points. Among six participants with baseline FA symptoms, all showed reductions in self-reported mYFAS 2.0 symptom counts. Four moved from mild to no symptoms, one from moderate to no symptoms, and one from severe to no symptoms. Across the full sample, the mean change was a reduction of 1.2 in the mYFAS 2.0 symptom counts per participant. Thematic analysis identified four interrelated psychological mechanisms: enhanced awareness of food–glucose relationships, increased accountability through shared tracking, motivation via gamified self-monitoring, and relief from cognitive burden associated with dietary uncertainty. **Conclusions**: Integrating CGM feedback into GMVs was associated with improvements in glycemic metrics and reductions in addictive-like eating symptoms in this pilot sample. These findings position CGM as a behavioral intervention tool that complements its traditional monitoring role and highlight the value of combining real-time biofeedback with group-based support in diabetes care.

## 1. Introduction

As of 2021, the Centers for Disease Control estimated that more than 38 million people in the United States were living with diabetes and over 97 million with prediabetes, underscoring the scale of dysglycemia as a public health challenge [1]. Type 2 Diabetes (T2D) management hinges on daily self-care behaviors, dietary choices, physical activity, medication use, and glucose monitoring, yet many individuals struggle to translate education into sustained behavior change [2]. Dysregulated eating, emotional eating, and loss of control around highly processed foods are strongly correlated with T2D [3].

In the face of the growing epidemic of diabetes, the introduction of continuous glucose monitors (CGMs) has changed how these patients view the direct relationship between their food intake and subsequent glucose readings [4]. Food addiction (FA) has emerged as a construct capturing compulsive, addictive-like responses to ultra-processed foods rich in refined carbohydrates and fats [5]. Population data indicates that prevalence of FA as measured by the modified Yale Food Addiction Scale (mYFAS) affects approximately 20% of adults, and with rates as high as 30% in T2D [6]. Although the FAconstruct remains an area of active debate, symptom-based measures such as the mYFAS 2.0 offer a pragmatic way to examine addictive-like eating behaviors relevant to diabetes self-management. FA symptoms, including cravings, diminished control, and continued overeating despite negative consequences, are associated with higher BMI, binge-eating pathology, and metabolic risk, and may help explain why some individuals experience disproportionate difficulty adhering to standard dietary recommendations [3,5,6]. Identifying and targeting FA-related mechanisms in T2D could therefore offer novel pathways to improving both behavioral adherence and glycemic outcomes.

CGMs have transformed how people with diabetes perceive the relationship between their daily choices and glucose fluctuations. Real-time continuous glucose monitoring systems provide near-continuous glycemic data, visual trend arrows, and summary metrics (e.g., time-in-range, glycemic variability) that can be used to guide both pharmacologic adjustments and lifestyle decisions [4,7]. Professional societies now recommend CGMs for selected adults with T2D to improve glycemic control and reduce hypoglycemia [8,9,10,11]. Recent qualitative and mixed-method studies in adults with T2D report that CGM can increase motivation, confidence in self-management, and perceived control by making glucose responses to meals and activities more visible and personally meaningful [12,13,14,15,16].

Beyond biomedical utility, CGMs can be conceptualized as a behavioral feedback tool embedded within self-regulation frameworks. Self-regulation and feedback-control frameworks propose that timely, interpretable feedback strengthens the perceived connection between behavior and physiological outcomes, thereby supporting self-monitoring and reinforcing adaptive health behaviors [17,18]. Early work has suggested that CGM-guided behavioral education and intermittent CGM use may prompt dietary modifications, increase awareness of physiological responses, and facilitate problem solving around glycemic excursions [19,20,21,22].

Parallel advances in digital health services offer additional context. Mobile health apps, Short Message Service (SMS)-based programs, and wearable activity trackers have been shown to improve self-management behaviors, self-efficacy, and in some cases glycemic outcomes in T2D by providing real-time or near-real-time feedback, goal tracking, and prompts [23,24,25,26,27]. For example, diabetes self-management apps and text-messaging interventions have improved hemoglobin A1c (HbA1c) and self-care behaviors by combining monitoring with tailored feedback and coaching [23,24]. Wearable activity trackers similarly leverage immediate feedback, self-monitoring, and goal setting to increase physical activity and support metabolic health that have explicitly examined how CGM, as a glucose-specific feedback modality, may influence addictive-like eating among adults with T2D [25,26].

The present mixed-methods study examines how CGM, when integrated into a structured group medical visit (GMV) program, may act as a feedback mechanism that strengthens awareness, accountability, and motivation around dietary behavior in adults with T2D. We assess changes in glycemic outcomes, patient activation, and FA symptoms (using the modified Yale Food Addiction Scale 2.0) and integrate these quantitative improvements with participants’ qualitative accounts of CGM-supported behavior change. By focusing on adults with T2D engaged in CGM-supported GMVs, this study aims to generate preliminary evidence on CGM’s potential to function as a behavioral intervention targeting both metabolic control and addictive-like eating.

## 2. Materials and Methods

This study was a secondary mixed-methods analysis of a previously implemented group medical visit (GMV) program that integrated CGM as a behavioral feedback tool for adults with T2D [27]. All participants used the Freestyle Libre 2 CGM that is worn on the upper arm. The Freestyle Libre 2 (Alemeda, CA, USA) is a 14-day, CGM system measuring glucose every minute, featuring optional real-time alarms, bluetooth connectivity for smartphone apps, a 40–400 mg/dL range, and water resistance, with a 1 h warm-up and measures 5 mm × 35 mm [28]. The GMV program was delivered over a 14-week period in a primary care setting and consisted of seven biweekly sessions, each lasting approximately 120 min. During these sessions, CGM data were reviewed collectively to facilitate goal setting and problem solving related to diet, physical activity, and self-management. A pragmatic mixed-methods approach was adopted, assuming quantitative and qualitative data offer complementary insights into behavioral change mechanisms. This study was approved by the institutional Investigational Review Board Human Protocol: IRB00058951.

The GMV program included seven sessions, each building on CGM feedback to guide learning and behavior change. Session 1 introduced participants to CGM use, nighttime hypoglycemia, and key glucose metrics while encouraging personal reflection through a motivation sheet and goal setting. From session 2 onward, participants’ weekly CGM tracings were reviewed, highlighting stable patterns and troubleshooting glycemic excursions with dietary swaps and physical activity. Session 2 focused on reactive hypoglycemia, individualized CGM targets such as the “No Hitter” goal with 100% time-in-range, diabetes complications, and the sugar content of common foods. Session 3 emphasized nutrition label reading, hidden sugars, dining strategies, macronutrient education, and the role of insulin resistance in metabolic syndrome. Session 4 shifted to physical activity, with participants writing personalized “exercise prescriptions.” Session 5 addressed the impact of stress and sleep on glucose, exploring mindfulness, stress relief, and sleep optimization. Session 6 introduced intermittent fasting (with a minimum 13 h fast and optional use of the Zero app for tracking) and reviewed cardiometabolic labs including cholesterol fractions and liver enzymes. Finally, session 7 consolidated all prior content, reviewed participant progress, and provided space for questions and discussion. All sessions were delivered by experienced providers including a certified diabetes care and education specialist and several family physicians.

### 2.1. Participants

The program enrolled 16 adults enrolled May 2021 to September 2021. For this analysis, we included participants with paired CGM summaries and paired psychosocial measures at baseline and follow-up, resulting in an analytic sample of 13 participants. Ten of these participants attended six or more sessions. The inclusion criteria for the original program required participants to be at least 18 years old, have a diagnosis of T2D, be willing to wear a CGM device, and complete baseline assessments.

### 2.2. Measures

CGM data were collected throughout the 14-week program [12]. For each participant, summary metrics were calculated for two standardized 14-day windows: Weeks 0–2 (baseline) and Weeks 12–14 (follow-up). These metrics consist of mean glucose in mg/dL, glycemic variability expressed as percent coefficient of variation (%CV), and percent time-in-range (TIR) defined as 70–180 mg/dL. We also recorded the percentage of time the CGM device was active. Summaries of mean glucose, %CV, and TIR were extracted when available for sensitivity analyses.

Psychosocial measures included the modified Yale Food Addiction Scale 2.0 (mYFAS 2.0), which was administered at baseline and follow-up to assess addictive-like eating behaviors [29]. We computed symptom counts and treated the change in symptom count as a primary psychological outcome. The Patient Activation Measure (PAM-13) was also administered at both time points, and we used both total scores and categorical levels (level 3 versus level 4) for descriptive profiling [30]. Health-related quality of life was assessed using the SF-12, which provided Physical Component Summary (PCS) and Mental Component Summary (MCS) scores at baseline and follow-up [31].

Qualitative data was obtained from open-ended survey questions administered at baseline and follow-up. These questions asked participants about their expectations and experiences with CGM, including perceived impacts on eating behavior, awareness, and daily management. These responses were used for thematic analysis and for constructing case vignettes.

### 2.3. Data Analysis

To standardize interpretation across outcomes, we defined improvement as a decrease in mean glucose and %CV, an increase in TIR, and a reduction in mYFAS 2.0 symptom count. For reporting purposes, we coded these changes so that positive values represented improvement. Changes in PAM, MCS, and PCS were calculated as follow-up minus baseline. We also created attendance strata (six or more sessions versus fewer than six) and defined psycho-behavioral phenotypes by crossing baseline mYFAS 2.0 status (any symptoms versus none) with PAM level (3 versus 4).

Given the small sample and non-normal distributions, we prioritized estimation using bootstrapped confidence intervals and non-parametric effect sizes (Cliff’s δ, Hedges’ g) over null-hypothesis testing [32,33,34,35,36]. We examined associations between engagement variables (sessions attended, percent CGM active at baseline and follow-up, and change in percent active) and improvements in psychosocial and CGM outcomes using Spearman’s rank correlation coefficients with bias-corrected and accelerated (BCa) 95% bootstrap confidence intervals with 5000 resamples. For transparency, we report percentile CIs in-text and provide BCa CIs in Supplementary Tables; both yielded the same inferences. Attendance contrasts were summarized using Cliff’s delta and Hedges’ g with small-sample correction, each accompanied by bootstrap confidence intervals [37]. Permutation *p*-values were calculated for mean differences but were reported only for completeness and not emphasized in interpretation. Phenotype profiles were presented as descriptive cell means with bootstrap confidence intervals and explicit sample sizes for transparency. Sensitivity analyses included re-estimating dose–response associations using 90-day CGM summaries and repeating analyses after 5% Winsorization of outcome distributions to assess robustness [38,39].

We conducted a reflexive thematic analysis of open-ended responses [40]. Two analysts independently reviewed responses and developed a codebook that included four themes: awareness from immediate feedback, accountability and engagement, motivation and gamification, and relief from finger-stick burden. Coding was iterative, and discrepancies were resolved through discussion. We created a theme-by-case matrix to summarize the presence or absence of each theme for every participant. For case vignettes, we selected participants whose quotations were particularly rich or illustrative and paired these with their quantitative change profiles.

**Figure 1:**
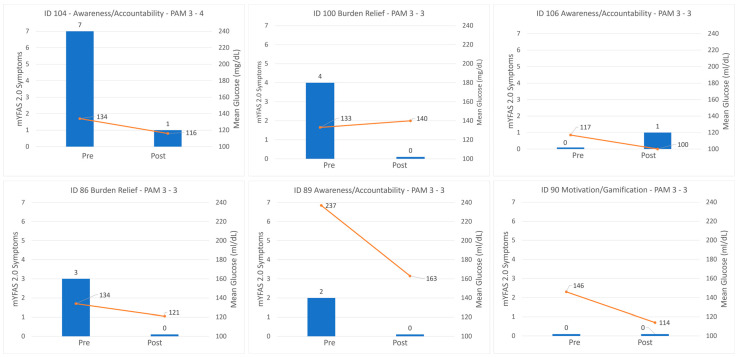
Case Vignettes: Psychological Themes and Glycemic Change Profiles. Note: This figure presents six participant vignettes (IDs 90, 89, 86, 106, 104, and 100) illustrating paired changes in mYFAS 2.0 symptom counts and CGM mean glucose from baseline (Weeks 0–2) to follow-up (Weeks 12–14), along with PAM level transitions. Each panel includes one verbatim quotation highlighting key psychological themes such as awareness, accountability, motivation, or burden relief. These vignettes exemplify the integration of patient voice with objective outcomes. One vignette (ID 90) illustrates a psychological mechanism (motivation/gamification) with no FA symptoms indicating CGM can help both those with and without FA symptoms.

Quantitative and qualitative findings were integrated through a theme-by-case joint display and case vignettes. Integration occurred at the interpretation and reporting stages, enabling triangulation of mechanisms (themes) with outcome changes (CGM, mYFAS). Figure 1 illustrates the mixed-methods flow.

## 3. Results

### 3.1. Participant Characteristics and Engagement

Of the 16 participants enrolled in the parent program, 13 had paired CGM and psychosocial data and were included in this analysis (participant characteristics are summarized in Table 1). The median age was 55 years old (25 to 69 years) and there were more males (62.5%) than female participants. The participants consisted of both Black (37.5%) and White individuals. Attendance was high, with a median of six sessions attended. Ten participants attended six or more sessions. The percentage time CGM was active was generally high at both baseline and follow-up.

### 3.2. Overall Changes from Baseline to Follow-Up

Participants demonstrated clinically meaningful improvements in CGM metrics. Individual pre-to-post changes in CGM and psychosocial measures are presented in Table 2. On average, mean glucose decreased by approximately 21 mg/dL, glycemic variability decreased by about two percentage points, and time-in-range increased by nearly nine percentage points. Regarding FA symptoms based on the mYFAS 2.0, seven participants reported no symptoms at either timepoint, while six participants showed reductions in symptom severity. Specifically, four participants moved from mild to no symptoms, one from moderate to no symptoms, and one from severe to no symptoms. Across all participants, the average reduction was 1.23 symptoms. Changes in PAM and SF-12 scores varied across individuals and are reported descriptively in the supplementary tables.

#### Dose–Response Associations

**Figure 2:**
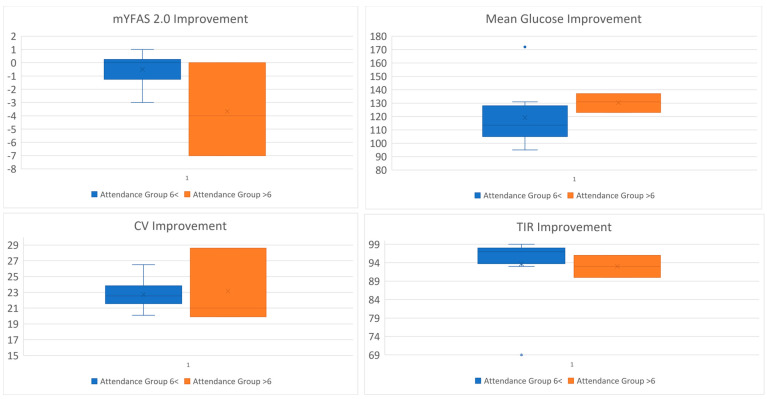
Exploratory Dose–Response Patterns by Attendance Strata. Note: This figure displays raincloud-style plots comparing improvements in food addiction symptoms, mean glucose, glycemic variability (%CV), and time-in-range (TIR) between participants attending six or more sessions versus fewer than six sessions. Individual data points are overlaid on distribution plots. Attendance contrasts are exploratory and interpreted descriptively; corresponding Spearman correlation coefficients with bootstrap confidence intervals are reported in Supplementary Table S1.

The following analyses are exploratory and intended to generate hypotheses for future study. Associations between engagement and outcomes were inconsistent and uncertain. The correlation between sessions attended and improvement in FA symptoms was approximately −0.49, with a bootstrap confidence interval ranging from −0.88 to 0.07. The correlation between sessions attended and improvement in time-in-range was approximately −0.43, with a confidence interval from −0.79 to 0.06. Attendance contrasts suggested that participants who attended fewer than six sessions had larger reductions in FA symptoms, but this finding is based on only three participants and should be interpreted with caution. Percent CGM active showed no stable association with improvements in psychosocial or CGM outcomes. These findings are presented visually and should be considered exploratory (Exploratory dose–response patterns by session attendance are displayed in Figure 2, with corresponding effect sizes shown in Supplementary Tables S1 and S2).

### 3.3. Psycho-Behavioral Phenotypes

**Figure 3:**
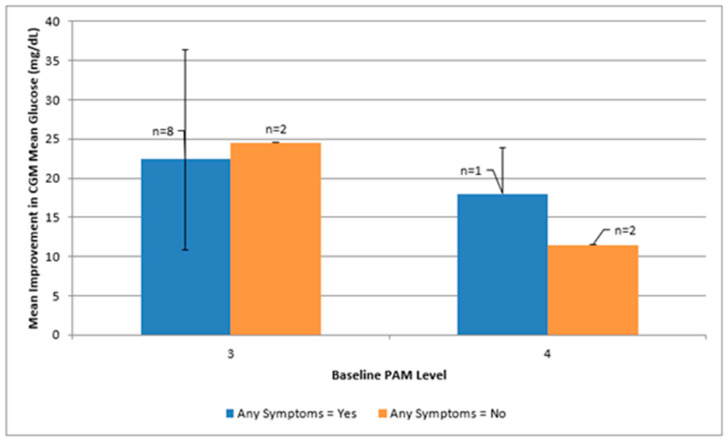
Phenotype Profiles: mYFAS 2.0 × PAM Level and Glycemic Improvement. Note: This figure illustrates mean improvements in CGM mean glucose across psycho-behavioral phenotype groups defined by baseline mYFAS 2.0 status (any symptoms vs. none) and PAM level (3 vs. 4). Error bars represent bootstrap 95% confidence intervals, and sample sizes are annotated for each cell. These profiles are descriptive only and are not intended to support subgroup inference. Sparse cells (n ≤ 2) should be interpreted with caution. This descriptive display highlights potential heterogeneity in response patterns for future hypothesis generation.

Baseline phenotype cells were unevenly distributed. Mean glycemic improvements across psycho-behavioral phenotypes defined by mYFAS 2.0 symptom burden and PAM level are shown in Figure 3 and summarized in Supplementary Table S3. The mYFAS 2.0 scale includes 11 possible symptoms, with higher scores indicating greater FA symptom burden. Participants presenting with FA symptoms and PAM level 3 (n = 8) demonstrated average improvements of approximately 22 mg/dL in mean glucose and 12 percentage points in time-in-range, along with an average reduction of about two symptoms on the mYFAS 2.0 scale; however, these descriptive patterns are based on small cell sizes and should not be interpreted as subgroup effects. Cells with two or fewer participants were too sparse for meaningful interpretation. Effect sizes for FA symptoms (mYFAS 2.0 ≥ 1 vs. 0) within PAM levels are provided in the supplementary tables but should be interpreted cautiously.

### 3.4. Mixed-Methods Case Vignettes

Case vignettes illustrate how psychological themes intersected with objective improvements (see Figure 1). Participants described the experience as engaging and motivating, reflecting gamification and positive reinforcement dynamics. For example, one participant (ID 104) described the program as “an excellent thing to be a part of… very helpful in monitoring my glucose” and experienced a reduction in seven self-reported FA symptoms, moving from the severe symptom range to no symptoms on the mYFAS 2.0, a decrease of 18 mg/dL in mean glucose, and an increase of 10 percentage points in time-in-range. Another participant (ID 90) stated, “It was sorta fun. It was like a numbers game… I liked doing what it took to drive them down” reflecting motivation and gamification and showed a 32 mg/dL improvement in mean glucose and a 14-point increase in time-in-range. These narratives, combined with quantitative data, underscore the role of awareness, accountability, and motivation in supporting behavior change.

### 3.5. Sensitivity Analyses

Re-estimating dose–response associations using 90-day CGM summaries and after Winsorization of outcome distributions did not materially change the interpretation. Engagement metrics were not reliably associated with improvements, and descriptive patterns remained consistent.

## 4. Discussion

The purpose of this study was to explore the psychological and behavioral changes associated with CGM-supported GMVs in adults with T2D, with particular attention to behaviors linked to FA. Within this small mixed-methods sample, participants demonstrated clinically meaningful improvements in glycemic outcomes over the 14-week program: an average 21 mg/dL reduction in mean glucose, approximately a full-percentage-point decrease in glycemic variability, and a nearly twelve-percentage-point increase in time-in-range (see Table 2). Regarding FA symptoms, seven of the 13 participants reported no symptoms at either baseline or follow-up, while the remaining six demonstrated improvement. Specifically, 4 participants moved from mild to no symptoms, one from moderate to no symptoms, and one from severe to no symptoms, based on mYFAS 2.0 scoring criteria (FA = 1 or fewer symptoms; mild = 2–3; moderate = 4–5; severe = 6 or more). Our findings are consistent with a behavioral feedback model in which CGM may support self-regulatory processes by increasing awareness and accountability. This immediacy may reinforce adaptive behavior through heightened awareness and accountability. Specifically, these findings suggest that integrating real-time CGM feedback into a structured, supportive group setting may be associated with concurrent metabolic improvements and reductions in addictive-like eating symptoms among adults managing T2D.

### 4.1. Interpretation and Mechanisms of Change

Although engagement metrics such as session attendance and CGM wear percentage did not show consistent dose–response relationships, the mixed-methods analysis revealed psychological mechanisms that co-occur with behavioral change. Qualitative and case-based data emphasize awareness, accountability, motivation and gamification, and relief from finger-stick burden as perceived catalysts of improvement (Table 3). Participants described how CGM feedback made glucose fluctuations visible and personally relevant transforming diabetes management from an abstract concept into a tangible, interactive process (illustrated in Figure 1). For some participants, monitoring became “a numbers game,” where seeing the direct impact of food and activity choices created a sense of mastery and enjoyment.

These mechanisms align with self-regulation and patient activation models, which posit that feedback enhances self-efficacy by strengthening the cognitive link between behavior and outcome [17,18], suggesting a possible role for CGM functioning as a real-time self-monitoring tool that provides immediate reinforcement and reduces the delay between action and consequence. Participants described increased awareness that coincided with reductions in compulsive or automatic eating behaviors. This process may be particularly valuable for individuals struggling with the behavioral rigidity, emotional eating, and loss of control that characterize food-addiction-like behaviors.

### 4.2. Comparison with Prior Literature

Previous studies have established the clinical utility of CGM for improving glycemic control, reducing hypoglycemia, and supporting medication titration in T2D [41]. Systematic reviews show that when combined with education and clinical follow-up, CGM use in T2D is associated with clinically meaningful reductions in HbA1c and increased time-in-range, even in non-insulin-treated populations [16,20]. However, most of this work has emphasized biomedical outcomes rather than the psychological processes that facilitate sustained self-management. Recent studies have begun to address this gap by examining how integrated behavioral interventions, such as therapeutic carbohydrate reduction combined with remote monitoring and behavioral support, can improve FA and binge eating symptoms, with 40.7% and 34.7% reductions from baseline, respectively [41]. Similarly, Unwin et al. (2022) demonstrated that whole-food, low-carbohydrate dietary interventions delivered through group visits can meaningfully reduce symptoms of ultra-processed FA even without CGM use [42]. In contrast, among 138 individuals who participated in 14 weeks of weekly group counseling focused on calorie restriction (1000–1200 kcal/day) through a meal replacement program combined with up to 175 min of weekly physical activity, FA symptoms improved by only 13.8% [43].

The present study extends this literature by explicitly linking CGM use to psychological constructs such as addictive-like eating, patient activation, and perceived behavioral control. Prior research on GMVs has shown that group-based education can improve self-efficacy and diabetes knowledge through peer learning and shared accountability [44]. By embedding CGM feedback into the GMV format, this study bridges these domains illustrating how social support and behavioral feedback can synergistically promote awareness and self-regulation [41]. These findings echo prior qualitative work reporting that CGM increases “body literacy” and emotional connection to physiological data, yet the current study uniquely quantifies concurrent improvements in food-addiction symptoms [7,13]. This integrated approach broadens the scope of CGM research beyond glucose numbers, emphasizing its psychological and behavioral relevance for complex metabolic conditions.

### 4.3. Clinical and Practical Implications

From a clinical perspective, the results highlight CGM’s potential to function as both a diagnostic and therapeutic instrument in behavioral diabetes and FA care [45,46,47]. Within GMVs, reviewing CGM tracings collectively allows participants to learn from one another’s data, normalize fluctuations, and co-construct behavioral strategies in real time. The emerging themes of awareness and accountability suggest that seeing one’s glucose response to food may counteract the denial or emotional disengagement that often perpetuates maladaptive eating [48]. Meanwhile, the gamification aspect viewing glucose improvement as a challenge or score to beat may introduce an intrinsically motivating element to self-management that sustains engagement even outside the clinical environment [49,50,51].

Importantly, observed reductions in FA symptom counts suggest that CGM-based feedback may be a useful context for exploring addictive-like eating behaviors in diabetes care. Unlike pharmacologic or purely educational interventions, CGM provides continuous experiential learning, transforming self-care into a feedback-driven habit rather than a cognitive task. Integrating such tools into primary-care GMVs may help clinicians target both the metabolic and psychological dimensions of diabetes care simultaneously, fostering more personalized, mechanism-based interventions.

### 4.4. Theoretical Considerations

The present findings contribute to emerging behavioral models that frame technology-enabled feedback as a mediator of self-regulation [52,53]. Specifically, they support a conceptual model in which psychological readiness (as reflected by the Patient Activation Measure) interacts with real-time behavioral feedback (from CGM) to enhance awareness, confidence, and adaptive action. Although the small sample precludes formal moderation testing, descriptive patterns suggest that participants with lower activation (PAM Level 3) and food-addiction symptoms derived particular benefit from CGM feedback. This potential interaction between internal readiness and external cues warrants further investigation as a pathway for tailoring interventions (see Figure 3).

Furthermore, these findings align with the broader literature on biofeedback, mindfulness, and interoceptive awareness, suggesting that making physiological states visible can transform health information into lived, actionable experiences [54,55]. CGM, therefore, may represent a digital health analog to mindfulness training encouraging reflective awareness of internal states, but through data visualization rather than meditation.

### 4.5. Limitations and Future Directions

Key strengths of this study include its mixed-methods design, which captured both quantitative outcomes and qualitative mechanisms, and its integration of validated psychosocial measures (mYFAS 2.0, PAM-13, SF-12) alongside objective CGM data. The inclusion of patient voice through verbatim quotations and case vignettes provides contextual richness often absent from metabolic studies, illustrating how patients interpret and internalize their data.

However, several limitations must be acknowledged. The small sample size limits statistical power and generalizability. The absence of a control group precludes causal inference, and improvements could partially reflect regression to the mean, increased clinical contact, or social desirability bias. Further, attendance contrasts and correlations were exploratory and unstable, emphasizing estimation rather than hypothesis testing. Additionally, participants willing to wear CGMs and attend GMVs may represent a more activated subset of patients, potentially inflating engagement effects. Thus, findings may not generalize to individuals with lower digital literacy or limited healthcare access. Finally, the short follow-up period prevents evaluation of long-term sustainability of both glycemic and psychosocial improvements.

Future research should confirm these preliminary findings in larger, controlled trials designed to isolate the mechanisms by which CGM influences behavioral change. Longitudinal follow-up is needed to determine whether reductions in food-addiction symptoms translate into sustained improvements in metabolic control and health-related quality of life. Incorporating ecological momentary assessment or digital logging could further elucidate how moment-to-moment feedback shapes food decisions and emotional responses.

Additionally, tailoring interventions by psycho-behavioral phenotype for example, matching CGM feedback intensity or coaching style to baseline activation or food-addiction severity may enhance engagement and efficiency. Finally, qualitative inquiry into how patients interpret CGM data across cultural, literacy, and motivational contexts could guide implementation strategies for diverse populations.

## 5. Conclusions

This exploratory mixed-methods study suggests that CGM functions not only as a biomedical monitoring tool but as a behavioral feedback system that promotes self-regulation and reduces addictive-like eating in adults with T2D. Integrating CGM into GMVs may operationalize feedback-based learning principles within routine care. Improvements in glucose control occurred alongside reductions in addictive-like eating behaviors and increased awareness and accountability, highlighting CGM’s potential as a feedback-driven behavioral intervention. While preliminary, these findings underscore the importance of integrating psychosocial support and self-regulation mechanisms into diabetes care. By merging objective data with patient reflection, CGM-supported GMVs may help patients move from reactive disease management toward proactive, empowered self-care addressing both the physiological and psychological roots of T2D. Future healthcare delivery models could leverage real-time biofeedback to support psychological readiness and behavioral adherence, improving outcomes across chronic disease management contexts.

## Figures and Tables

**Table 1:** Participant Characteristics and Baseline Measures.

Participant ID	Baseline Weight (lbs)	Baseline HbA1c	Baseline PAM		Baseline SF-12	
			Score	Level	PCS	MCS
86	195.4	7.8	60.6	3	41.0	50.8
87	226	7.1	63.1	3	43.6	53.9
88	342.4	6.6	67.8	3	50.8	49.9
89	209.4	7.5	70.2	3	39.3	45.5
90	246	6.9	63.1	3	57.1	57.4
91	297.8	6.6	84.8	4	54.6	57.5
99	246.4	6.6	58.1	3	34.3	37.7
100	232	6.8	65.5	3	51.5	56.1
102	254.2	7.8	65.5	3	54.4	55.2
103	183.4	7.3	84.8	4	48.3	49.2
104	274.8	6.8	65.5	3	41.0	44.9
105	202.2	7	72.5	4	52.3	54.2
106	221.2	7.3	60.6	3	51.8	49.4

This table summarizes demographic and clinical characteristics of the analytic sample), including baseline HbA1c, weight, and baseline psychosocial scores (MYFAS, PAM, SF-12).

**Table 2:** Pre-to-Post Changes in Glycemic and Psychosocial Outcomes.

Participant ID	Improvement in Mean Glucose (mg/dL)	Improvement in %CV	Improvement in %TIR	Improvement in mYFAS 2.0 Symptoms	Improvement in PAM Score	Improvement in SF-12 MCS	Improvement in SF-12 PCS
86	+13	+0.8	+5	+3	0	+2.2	+9.8
87	+9	+1.0	+8	+1	+13.1	+3.4	+3.9
88	+18	+1.2	+7	0	+4.7	+1.0	+4.3
89	+74	+0.6	+69	+2	+2.5	+5.4	+6.2
90	+32	+1.5	+14	0	0	+1.8	+3.2
91	+22	+0.9	+10	0	−3.9	+2.0	+2.5
99	+15	+1.1	+6	0	+2.5	+1.7	+3.1
100	+20	+1.3	+8	+4	+2.3	+2.8	+4.0
102	+17	+1.0	+9	0	+7.0	+3.5	+3.8
103	+11	+0.7	+5	+1	−19.3	+2.2	+3.6
104	+18	+1.4	+10	+7	+9.5	+4.1	+4.9
105	+16	+1.2	+8	+1	+8.4	+3.9	+4.3
106	+14	+1.0	+6	0	−5.0	+2.5	+3.7

This table reports descriptive statistics for changes from baseline to follow-up in CGM metrics (mean glucose, %CV, TIR) and psychosocial measures (mYFAS 2.0 symptom count, PAM score, SF-12 PCS and MCS). Positive values indicate improvement for all outcomes.

**Table 3:** Themes and Exemplar Quotations from Qualitative Analysis.

Theme	Exemplar Quote
Awareness from immediate feedback	“with the CGM it brought about an awareness to be vigilant about food and drink intake”
Accountability and engagement	“it helps to keep me accountable to myself because I can see the numbers in almost real time”
Motivation and gamification	“it was sorta fun. It was like a numbers game… I liked doing what it took to drive them down”
Relief from finger-stick burden	“the CGM ensures far more frequent glucose tests as opposed to finger pricks”

Quotations are de-identified and illustrate participants’ perceived mechanisms of change.

## Data Availability

The original contributions presented in this study are included in the article/Supplementary Material. Further inquiries can be directed to the corresponding author.

## Supplementary Materials

The following supporting information can be downloaded at: https://www.mdpi.com/article/10.3390/healthcare14040420/s1, Table S1: Dose–Response Associations Between Engagement and Outcomes; Table S2: Attendance Contrasts for Improvements in Outcomes; Table S3: Phenotype Profiles and Effect Sizes.

## Abbreviations

The following abbreviations are used in this manuscript:

CGM	Continuous Glucose Monitoring
FA	Food Addiction
GMV	Group Medical Visit
TIR	Time-in-Range
mYFAS 2.0	modified Yale Food Addiction Scale 2.0
PAM-13	Patient Activation Measure 13
PCS	Physical Component Summary
MCS	Mental Component Summary
