# Supplementary material for: Psychological Aspects and Implications of Food Addiction and Glucose Control in Type 2 Diabetes: A Pilot Mixed-Methods Study
Source: Healthcare (Basel). 2026 Feb 7;14(4):420. doi: 10.3390/healthcare14040420 (PMC12941053; doi:10.3390/healthcare14040420)
Supplement: Supplementary file 1 [file healthcare-14-00420-s001.zip › healthcare-4091498-supplementary.pdf]

Supplemental Materials

Supplementary Table S1. Dose–Response Associations Between Engagement and Outcomes.

Predictor	Outcome	N	Spearman ρ	95% CI Low	95% CI High
Sessions	Δ mYFAS 2.0 Symptoms	13	−0.49	−0.88	+0.07
Sessions	Δ Mean Glucose	13	−0.12	−0.55	+0.31
Sessions	Δ Mean TIR	13	−0.43	−0.079	+0.06
% CGM Active	Δ mYFAS 2.0 Symptoms	13	−0.31	−0.79	+0.32
% CGM Active	Δ Mean Glucose	13	−0.15	−0.60	+0.43

Note: This table provides Spearman correlation coefficients with bootstrap 95% confidence intervals for associations between engagement metrics (sessions attended, percent CGM active) and improvements in psychosocial and glycemic outcomes. These analyses are exploratory and intended for estimation rather than hypothesis testing.

Supplementary Table S2. Attendance Contrasts for Improvements in Outcomes.

Outcome	n High	n Low	Cliff's δ	95% CI Low	95% CI High	Hedges' g
Δ mYFAS 2.0 Symptoms	10	3	−0.60	−0.95	+0.05	−0.72
Δ Mean Glucose	10	3	−0.10	−0.50	+0.30	−0.15

Note: This table reports Cliff's delta and Hedges' g effect sizes with bootstrap confidence intervals for differences in outcome improvements between participants attending six or more sessions versus fewer than six sessions. Permutation p-values are included for completeness but are not emphasized in interpretation.

Supplementary Table S3. Phenotype Profiles and Effect Sizes.

mYFAS 2.0 Any	PAM Level	n	Δ mYFAS 2.0 Symptoms	Δ Mean Glucose	Δ %TIR
True	3	8	+2.13	+22.4	+12.3
False	3	2	−0.5	+24.5	+9.5
False	4	2	−0.5	+11.5	−1.5
True	4	1	+7.0	+18.0	+10.0

Note: This table summarizes mean improvements in glycemic and psychosocial outcomes across phenotype groups defined by baseline mYFAS 2.0 status and PAM level. Effect sizes (Hedges' g and Cliff's delta) for mYFAS 2.0-any versus none within PAM strata are provided with cautionary interpretation due to small sample sizes.
